# Supplementary material for: The Stringent Stress Response Controls Proteases and Global Regulators under Optimal Growth Conditions in Pseudomonas aeruginosa
Source: mSystems. 2020 Aug 4;5(4):e00495-20. doi: 10.1128/mSystems.00495-20 (PMC7406228; doi:10.1128/mSystems.00495-20)
Supplement: TABLE S1 [file mSystems.00495-20-st001.docx]

***SI Appendix;* Table S1A: Bacterial strains used in this study**

| **Strains** | **Relevant characteristics or genotype^a^** | **Reference or source** |
| --- | --- | --- |
| ***Escherichia coli*** | | |
| XL1-Blue | *recA*1 *endA*1 *gyrA*96 *thi*-1 *hsdR*17(rK- mK+) *supE*44 *relA*1 *lac*, [F′ *proAB lacIq* ZΔM15Tn10(Tetracycline resistance)] | Stratagene |
| ***Pseudomonas aeruginosa* PAO1 strains** | | |
| WT | Laboratory wild type strain | (1) |
| Δ*relA*Δ*spoT* | Δ*relA*Δ*spoT* double deletion mutant | (2) |
| Δ*relA*Δ*spoT*/*relA*^+^ | Δ*relA*Δ*spoT* deletion mutant chromosomally complemented with the *relA* gene including its promoter region | (2) |
| PAO1.Δ*relA*Δ*spoT*/*spoT*^+^ | Δ*relA*Δ*spoT* deletion mutant chromosomally complemented with the *spoT* gene including the *rpoZ*-*spoT* promoter region | (2) |
| PAO1/pUCP22.relA-Pro.mCherry | PAO1 carrying *relA* promoter fusion to *mCherry* | This study |
| PAO1/pUCP22.cysM-Pro.mCherry | PAO1 carrying *cysM* promoter fusion to *mCherry* | This study |
| PAO1/pUCP22.cysM-relA-Pro.mCherry | PAO1 carrying *cysM-relA* promoter fusion to *mCherry* | This study |
| PAO1/pUCP23.rpoZ-Pro.egfp | PAO1 carrying *rpoZ* promoter fusion to *egfp* | This study |
| PAO1/pHERD20T.*relA* | PAO1 carrying *relA* overexpression plasmid | This study |
| PAO1/pHERD20T.*spoT* | PAO1 carrying *spoT* overexpression plasmid | This study |
| PAO1/pHERD20T.*relA-YFP* | PAO1 carrying *relA-YFP* overexpression plasmid | This study |
| PAO1/pHERD20T.*spoT-GFP* | PAO1 carrying *spoT-GFP* overexpression plasmid | This study |
| PAO1.Δ*argB* | Δ*argB* deletion mutant | This study |
| PAO1.Δ*argB/*pUCP22.relA-Pro.mCherry | PAO1.Δ*argB* carrying *relA* promoter fusion to *mCherry* | This study |
| PAO1.Δ*argB/*pUCP22.cysM-Pro.mCherry | PAO1.Δ*argB* carrying *cysM* promoter fusion to *mCherry* | This study |
| PAO1.Δ*argB*/pUCP22.cysM-relA-Pro.mCherry | PAO1 carrying *cysM-relA* promoter fusion to *mCherry* | This study |

***SI Appendix;* Table S1B: Plasmids used in this study**

| **Plasmids** | **Phenotype** | **Reference** |
| --- | --- | --- |
| pEX18Gm | Gm^r^, pEX18Gm sucrose suicide vector carrying the *cysM*-*mCherry* promoter fusion | (3) |
| pEX18Gm.argB-ko | Gm^r^, pEX18Gm sucrose suicide vector carrying the *argB* knockout alleles | This study |
| pHERD20T | Ap^r^*, Escherichia-Pseudomonas* shuttle vector carrying *araC*-P_BAD_ cassette | (4) |
| pUCP22 | Ap^r^Gm^r^, Broad-host-range vector | (5) |
| pUCP23 | Ap^r^Gm^r^, Broad-host-range vector | (5) |
| pUCP23.mCherry | Ap^r^Gm^r^, pUCP23 carrying the *mCherry* red fluorescent protein gene | (6) |
| pUCP22.relA-Pro.mCherry | Ap^r^Gm^r^, pUCP22 carrying the *relA*-*mCherry* promoter fusion | This study |
| pUCP22.cysM-Pro.mCherry | Ap^r^Gm^r^, pUCP22 carrying the *cysM*-*mCherry* promoter fusion | This study |
| pUCP22.cysM-relA-Pro.mCherry | Ap^r^Gm^r^, pUCP22 carrying the *cysM-relA*-*mCherry* promoter fusion | This study |
| pHERD20T.*relA* | Ap^r^, pHERD20T carrying the *relA* gene from PAO1 und P_BAD_ | This study |
| pHERD20T.spoT | Ap^r^, pHERD20T carrying the *spoT* gene from PAO1 und P_BAD_ | This study |
| pBAD24.YFP | Ap^r^, pBAD24 carrying the *yfp* gene and N-terminal linker sequence | (7) |
| pBBR1.egfp.TIR | Cm^r^, pBBR1MCS carrying *egfp*-T_0_ cassette in pBBR1MCS in opposite orientation of P_lac_ | (8) |
| pHERD20T.*relA-YFP* | Ap^r^, pHERD20T carrying the *relA* gene without stop codon fused to linker sequence and *yfp* | This study |
| pHERD20T.spoT-GFP | Ap^r^, pHERD20T carrying the *spoT* gene without stop codon fused to linker sequence and *egfp* | This study |

^a^ Antibiotic resistance: Gm^r^, gentamicin; Ap^r^, ampicillin / carbenicillin; Cm^r^, chloramphenicol

***SI Appendix;* Table S1C: PCR primers used in this study**

| **Primer** | **Sequence** |
| --- | --- |
| **PCR primers** |  |
| argB_up_fwd | AGAGGATCCGTGGAAGACCGGCCACTC |
| argB_up_rev | GTGGCGGGCGATCAGTGAGGGGAGCTCCGACATGTC |
| argB_down_fwd | GACATGTCGGAGCTCCCCTCACTGATCGCCCGCCAC |
| argB_down_rev | TGCAAGCTTCCAGCCACTGGCTGGACA |
| argB_outA | ATGCTGTTCGCCAAGGACGT |
| argB_outB | ATCGAACTCATCCATGCCGC |
| aprA-up_fwd | AAGTACGACTACCTCACGGC |
| aprA-up_rev | TGTCAGCGCGTCAGACGACGATGTCCAATGTCCACAGGTTTC |
| aprA-down_fwd | GAAACCTGTGGACATTGGACATCGTCGTCTGACGCGCTGACA |
| aprA-down_rev | ATGATCGGTCGCTTCGTGGT |
| aprA_outA | TTACGACCTGGACGACAAGG |
| aprA_outB | GACCCATGAGATCGAAGGCA |
| relA-Pro_rev/mCherrry_fwd | ATCCTCCTCGCCCTTGCTCACCATCTTGCCTACCCTTTACCACG |
| relA-pro_fwd(Xba) | CATTCTAGAGATGCCTGCGTAATCCGA |
| relA-Pro_rev/mCherrry_fwd | CGTGGTAAAGGGTAGGCAAGATGGTGAGCAAGGGCGAGGAGGAT |
| mCherry_rev_t0(KpnI-ApaI) | CATGGTACCGGGCCCTGGACTCACAAAGAAAAAACGC |
| cysM-Pro_fwd(Xba) | AGTTCTAGAACCACCTCGTTCTGATCGCC |
| cysM-pro_rev(BamHI) | TCAGGATCCGAGGCACTCGTTCGGCACTGACG |
| cysM-pro_rev(SpeI) | CATACTAGTGAGGCACTCGTTCGGCACTGACG |
| rpoZ-Pro_fwd | CCAGGGCCCGAGCTCGTCTGCTCGCCTAAATCGG |
| rpoZ-Pro_rev/egfp_fwd | CACTTTTCGTTACGAGGAACACCATGCTGAGCAAGGGCGAGGA |
| egfp_fwd/rpoZ-Pro_rev | TCCTCGCCCTTGCTCAGCATGGTGTTCCTCGTAACGAAAAGTG |
| egfp_rev | AAGCTGGGTACCAACGGTGG |
| relA_oe_fwd | TCTAGAATGGTACAGGTGAGAGCGCA |
| relA_oe_rev | AAGCTTGGGTATCTCGGGTCTTCAG |
| spoT_fwd | TTGCCGGGCATAGACGCCTT |
| spoT_rev | GCCAAGCTTGGAAAAAGCGGGTCAGCTACG |

**References**

1. R. E. W. Hancock, A. M. Carey, Outer membrane of *Pseudomonas aeruginosa*: heat- 2 mercaptoethanol-modifiable proteins. *J Bacteriol* **140**, 902-910 (1979).
2. D. Pletzer, H. Wolfmeier, M. Bains, R. E. W. Hancock, Synthetic peptides to target stringent response-controlled virulence in a *Pseudomonas aeruginosa* murine cutaneous infection model. *Front Microbiol* **8**, 1867 (2017).
3. D. Pletzer, Y. Braun, H. Weingart, Swarming motility is modulated by expression of the putative xenosiderophore transporter SppR-SppABCD in *Pseudomonas aeruginosa* PA14. *Antonie Van Leeuwenhoek* **109**, 737-753 (2016).
4. D. Qiu, F. H. Damron, T. Mima, H. P. Schweizer, H. D. Yu, PBAD-based shuttle vectors for functional analysis of toxic and highly regulated genes in *Pseudomonas* and *Burkholderia* spp. and other bacteria. *Appl Environ Microbiol* **74**, 7422-7426 (2008).
5. S. E. West, H. P. Schweizer, C. Dall, A. K. Sample, L. J. Runyen-Janecky, Construction of improved *Escherichia-Pseudomonas* shuttle vectors derived from pUC18/19 and sequence of the region required for their replication in *Pseudomonas aeruginosa*. *Gene* **148**, 81-86 (1994).
6. C. L. Berry *et al.*, Chemical and biological characterization of sclerosin, an antifungal lipopeptide. *Can J Microbiol* **58**, 1027-1034 (2012).
7. M. Berger *et al.*, Genes on a wire: The nucleoid-associated protein HU insulates transcription units in *Escherichia coli*. *Sci Rep* **6**, 31512 (2016).
8. A. Burse, H. Weingart, M. S. Ullrich, The phytoalexin-inducible multidrug efflux pump AcrAB contributes to virulence in the fire blight pathogen, *Erwinia amylovora*. *Mol Plant Microbe Interact* **17**, 43-54 (2004).
